# Clinical characteristics and prescriptions associated with a 2-year course of rapid cycling and euthymia in bipolar disorder: a multicenter treatment survey for bipolar disorder in psychiatric clinics

**DOI:** 10.3389/fpsyt.2023.1183782

**Published:** 2023-05-17

**Authors:** Chikashi Takano, Masaki Kato, Naoto Adachi, Yukihisa Kubota, Takaharu Azekawa, Hitoshi Ueda, Kouji Edagawa, Eiichi Katsumoto, Eiichiro Goto, Seiji Hongo, Kazuhira Miki, Takashi Tsuboi, Norio Yasui-Furukori, Atsuo Nakagawa, Toshiaki Kikuchi, Koichiro Watanabe, Toshihiko Kinoshita, Reiji Yoshimura

**Affiliations:** ^1^Department of Neuropsychiatry, Kansai Medical University, Osaka, Japan; ^2^Japanese Society of Clinical Neuropsychopharmacology, Tokyo, Japan; ^3^Japanese Association of Neuro-Psychiatric Clinics, Tokyo, Japan; ^4^Department of Neuropsychiatry, Kyorin University School of Medicine, Tokyo, Japan; ^5^Department of Psychiatry, Dokkyo Medical University, Tochigi, Japan; ^6^Department of Neuropsychiatry, Keio University School of Medicine, Tokyo, Japan; ^7^Department of Psychiatry, University of Occupational and Environmental Health, Fukuoka, Japan

**Keywords:** bipolar disorder, rapid cycling, euthymia, real world, mood stabilizer, outpatient, cross-sectional study, antidepressants

## Abstract

**Objective:**

In patients with bipolar disorder (BD), rapid cycling (RC) presents a risk for a more severe illness, while euthymia (EUT) has a better prognosis. This study focused on the progression of RC and EUT, which are contrasting phenomenology, and aimed to clarify the influence of patient backgrounds and prescription patterns on these different progressions, using a large sample from the first and second iterations of a multicenter treatment survey for BD in psychiatric clinics (MUSUBI).

**Methods:**

In the cross-sectional study (MUSUBI), a questionnaire based on a retrospective medical record survey of consecutive BD cases (*N* = 2,650) was distributed. The first survey was conducted in 2016, and the second one in 2017. The questionnaire collected information on patient backgrounds, current episodes, and clinical and prescribing characteristics.

**Results:**

In the first survey, 10.6% of the participants had RC and 3.6% had RC for two consecutive years, which correlated with BP I (Bipolar disorder type I), suicidal ideation, duration of illness, and the use of lithium carbonate and antipsychotic medications. Possible risk factors for switching to RC were comorbid developmental disorders and the prescription of anxiolytics and sleep medication. Moreover, 16.4% of the participants presented EUT in the first survey, and 11.0% presented EUT for two consecutive years. Possible factors for achieving EUT included older age; employment; fewer psychotic symptoms and comorbid personality disorders; fewer antidepressants, antipsychotics, and anxiolytics, and more lithium prescriptions.

**Conclusion:**

RC and EUT generally exhibit conflicting characteristics, and the conflicting social backgrounds and factors contributing to their outcomes were distinctive. Understanding these clinical characteristics may be helpful in clinical practice for management of patients with BD.

## 1. Introduction

Bipolar disorder (BD) is a pervasive, severe, chronic, recurrent, and potentially lethal mental illness that typically manifests as episodes of either manic or depressive polarity, which can last from days to weeks and even months, followed by periods of either remission or the opposite polarity. According to the World Health Organization, this disorder affects approximately 60 million individuals globally, making it the sixth most widespread and rapidly increasing disease worldwide. The lifetime prevalence of the disorder is estimated to be 3% (1, 2); however, the prevalence rates among countries range from 0.2% to 4%. Depressive episodes of BD are often misdiagnosed as depression, with previous reports indicating that 20% of patients diagnosed with depression subsequently received a changed diagnosis of BD. The disorder is also one of the leading causes of disability worldwide, with patients experiencing significant disability for most of their lives, particularly due to depressive symptoms (3–5). Additionally, it has been reported that the rates of suicide and self-harm for this disorder were over 10% and 30–40%, respectively, during their lifetime. While the course of BD varies from person to person, the number of mood episodes has been correlated with various clinical and neurophysiological parameters, such as cognitive function, quality of life, response to pharmacological and socio-psychological treatment, and brain structure (6–8). This is consistent with the paradigm that BD is not only cyclical but also progressive (6, 9). The concept of “Rapid Cycling (RC),” pertaining to four or more mood episodes in a year, was introduced by Dunner and Fieve in the early 1970s as an indication of the course of BD (10). RC is reported to affect 5–30% of patients with bipolar disorder, with a prevalence of approximately 10% reported in a large-scale survey in Japan (11). It has been recognized that patients with RC tend to respond poorly to medication (12), especially lithium (13). More recent research has indicated that these patients may not respond to any treatment and are at a higher risk for poor prognosis, including prolonged treatment, decreased Global Assessment of Functioning (GAF) scores (14, 15), and an increased risk of suicide attempts (12, 14, 16–18). In addition, previous studies have found that patients with RC demonstrate a higher total number of episodes and hospitalizations (19–21), shorter symptom-free periods between episodes (22), more severe disability (15, 23–27), and a higher risk of suicide (23). It has also been suggested that, similar to epilepsy, RC may increase over time (9, 22), and that repeated episodes may shorten the cycle and cause RC, a theory known as the “kindling hypothesis.” In contrast, approximately 20% of patients with BD who recover from mood episodes maintain recovery for approximately 60 weeks (28). Given the importance of distinguishing between RC and long-term asymptomatic BD, which is associated with a good prognosis, and the impact of repeated relapses on the threshold for the occurrence of episodes and the kindling phenomenon, there is a need for large-scale clinical trials examining the epidemiology, background, clinical and social characteristics, and psychotropic drug prescribing patterns of patients with RC and those with long-term asymptomatic BD, as well as changes in these phenotypes over time. The MUlticenter treatment SUrvey on BIpolar disorder in Japanese psychiatric clinics (MUSUBI) is the largest nationwide cross-sectional survey to date on the status of patients with BD in Japan (11, 29–38). In 2020, we identified patients with conflicting phenotypes of current RC and 1-year euthymia (EUT) from the MUSUBI first survey data, and reported differences in patient backgrounds, current episodes, clinical features, and prescribing patterns. However, because it was based on data from a single cross-sectional survey, we were unable to evaluate RC and EUT over time, the clinical features associated with the course of the disease, or its association with medications. In the present study, we focused on the progression of RC and EUT, which are conflicting phenotypes, and aimed to clarify the influence of patient backgrounds and prescription patterns on these different progressions, using the results of a 2-year follow-up study on patients who developed RC and EUT in 2016.

## 2. Methods and materials

### 2.1. Participants and study design

The Japanese Association of Psychiatry and Neurology Clinics (JAPC) and the Japanese Society of Clinical Neuropharmacology have initiated a joint study called “MUSUBI” to gather evidence on the treatment of BD in Japan. The design and methodology of MUSUBI have been previously described (32, 37). It is a cross-sectional study comprising a questionnaire survey administered to 176 clinics affiliated with the JAPC (11% of all JAPC members). The subjects were patients aged 18–75 years who were diagnosed with BD by their attending physicians according to the International Classification of Diseases and Related Health Problems, Tenth Edition (ICD-10) (World Health Organization) (39) during the study period. The first survey was conducted from September 1 to October 31, 2016, and the second survey was conducted from September 1 to December 31, 2017, with consecutive assessments of the same patients. The attending physician was asked to complete a questionnaire based on a retrospective chart survey of the patients with BD. The questionnaire included questions on patient characteristics, including age, age at onset, sex, height, weight, education, occupation, comorbidities, current conditions, course of illness, duration of current episode, subcategories, the GAF score, suicidal ideation, psychotic symptoms, and medication details. Patients with serious physical illnesses or those deemed unfit to participate by a psychiatrist were excluded. RC was defined as at least four mood episodes that met the criteria for mania, hypomania, or major depression in the previous 12 months. EUT was defined as asymptomatic presentation (no manic symptoms, depression, or suicidal ideation) with no recurrence for at least 12 months. We extracted data on RCs and EUTs for each survey and analyzed their frequencies, transitions, associated patient backgrounds, and prescription patterns. The transitions for RC and EUT were analyzed in four ways (1–1, 1-0, 0-0, 0–1), with 1 indicating presence and 0 as absence, for example, 1–1 RC (RC at the first survey, RC at the second survey) and 1–0 EUT (EUT at the first survey, non-EUT at the second survey), to determine which patient background and prescribed medications were associated with each outcome. The medications included antidepressants, antipsychotics, mood stabilizers, sleep medications, and anxiolytics. Lithium carbonate, valproic acid, carbamazepine, and lamotrigine were included as mood stabilizers in the analysis.

### 2.2. Statistical analysis

The SPSS® software version 27 (IBM Corp., Armonk, NY, USA) was used for analysis. Fisher’s exact test or analysis of variance was used for each factor in univariate analysis. Multivariate logistic regression analysis was then performed for each factor to avoid confounding effects. In this analysis, RC or EUT at the time of the second survey was used as the dependent variable, and sex, age, age at onset, body mass index (BMI), occupational status, GAF score (>60), education (college or higher), suicidal ideation, psychotic symptoms, comorbidity of personality disorders, neurodevelopmental disorders (autism spectrum disorder and Attention-Deficit/Hyperactivity Disorder (ADHD)), alcohol or substance abuse, and physical disability at the time of the first survey were used as candidate independent variables. Factors that showed alpha levels <0.1 in the univariate analysis were included in the model using a stepwise method (F-entry probability: 0.05; removal: 0.1). Adjusted alpha levels <0.004 (0.05/number of candidate independent variables) and < 0.05 were considered significant in univariate analysis and logistic regression analysis, respectively. To understand the characteristics of psychiatric comorbidities associated with RC and EUT, the regression analysis used personality disorders, neurodevelopmental disorders, and alcohol and substance abuse comorbidity rates rather than the total psychiatric comorbidity rates. Furthermore, improvements in the GAF score were highly correlated with RC and EUT, and GAF was not included in the logistic regression analysis. The impact of medications on the outcomes was subsequently analyzed using propensity score matching as an exploratory study. Propensity scores were calculated using a logistic regression model with the following factors: sex; age; education; BMI, age at onset; psychiatric comorbidity; comorbidities; psychotic symptoms; suicidal ideation; GAF score; severity of depressive symptoms; severity of manic symptoms, and whether the patient received mood stabilizers, antidepressants, antipsychotics, anxiolytics, or sleep medications.

### 2.3. Ethics statement

This study was conducted in accordance with the Declaration of Helsinki and the Japanese “Ethical Guidelines for Epidemiological Research.” Before the study began, the research protocol was reviewed and approved by the Institutional Review Boards of the Japan Neuropsychiatric Clinics Association and the Ethics Committee of Kansai Medical University. As this study was a retrospective medical record survey, relevant information was disclosed so that patients could opt out if they so wished. A study-specific subject identification number was assigned to each patient to ensure linkable anonymity in order to protect the patients’ personal information.

## 3. Results

**Figure 1:**
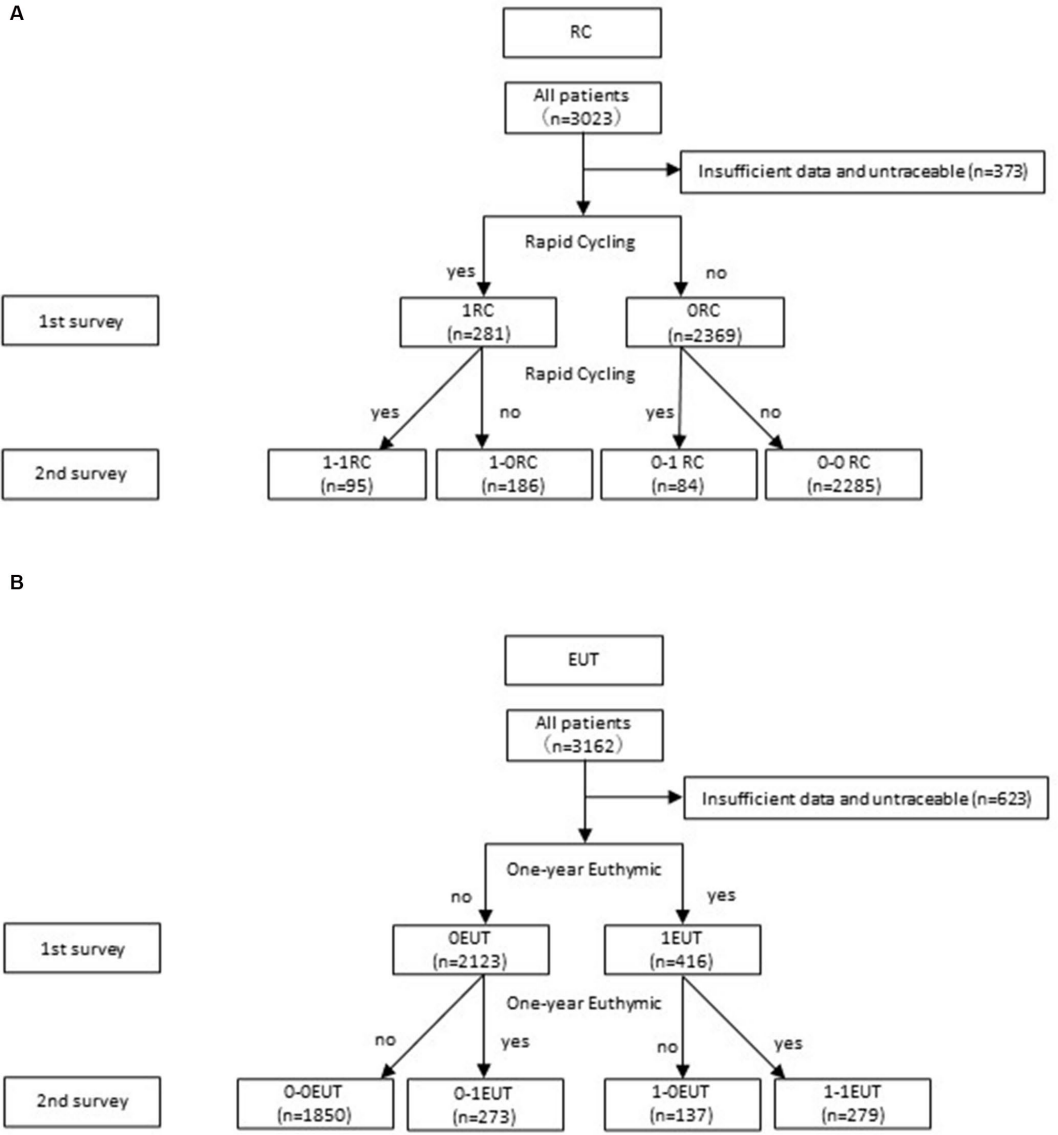
**(A)** Flow chart of study sample selection of RC. RC; Rapid Cycling, 1RC; RC at the 1st survey, ORC; non-RC at the 1st survey, 1-1RC; RC at the 1st and 2nd survey, 1-ORC; RC at the 1st survey and non-RC at the 2nd, 0-1RC; non-RC at the 1st survey and RC at the 2nd, 0-ORC; non-RC at the 1st and 2nd survey. **(B)** Flow chart of study sample selection of EUT. EUT; one-year euthymic, OEUT; non-EUT at the 1st survey, 1EUT; EUT at the 1st survey, O-OEUT; non-EUT at the 1st and 2nd survey, 0-1EUT; non-EUT at the 1st survey and EUT at the 2nd, 1–0EUT; EUT at the 1st survey and non-EUT at the 2nd, 1-1EUT; EUT at the 1st and 2nd survey.

Questionnaire results were collected from 3,213 patients with BD who completed both the first and second surveys at 176 clinics. We excluded incompatible cases and finally analyzed 3,084 patients overall: 2,539 with EUT and 2,650 with RC (Figures 1A,B). The demographics of RC and EUT, respectively, were as follows: 45.7 and 45.6% (female); mean age, 50.26 ± 13.86 (Standard Deviation: SD) years and 50.32 ± 13.88 (SD) years; mean age of onset, 34.72 ± 12.49 (SD) years and 34.73 ± 12.50 (SD) years; BP I percentage, 35.1%, and 35.3%.

### 3.1. Rapid cycling

RCs were observed in 10.6% (281/2,650) of patients in the first survey, whereas 3.6% (95/2,650) presented RC for two consecutive years. Out of those with RC in the first survey, 33.8% (95/281) continued presenting in the following year. Additionally, 53.1% (95/179) of those with RC in the second survey had also presented RC in the previous year. Of the non-RC-presenting individuals in the first survey, 3.5% (84/2,369) showed RC and 86.2% (2,285/2,369) never presented RC within the following 2 years. Patients who presented RC in the first survey significantly maintained the presentation in the following year (odds ratio (OR) [95% confidence interval (CI)] = 13.9 [10.0–19.3], *p* < 0.0001).

#### 3.1.1. Outcome of patients with RC in the first survey

The outcomes of patients who presented RC in the first survey were examined in the second (Table 1). Univariate analysis showed significant differences in the BP subtype (*p* < 0.001). Multivariate logistic regression analysis showed that 1–1 RC had higher BP I (OR [95% CI] = 2.217 [1.091–3.805], *p* = 0.004) and suicidal ideation (OR = 2.018 [1.062–3.8347], *p* = 0.032) and longer disease duration (OR = 1.026 [1.001–1.052], *p* = 0.042) than 1–0 RC.

**Table 1:** Impact of clinical characteristics of 1RC.

Variables	1–0RC	1-1RC	Stat	*p*	*p* multivariate	OR (95% CI)
	*n* = 95	*n* = 186				
*n* (%)			*χ* ^2^			
Female	35.5	35.8	0.003	0.960		
Bipolar I	38.5	59.8	11.040	**<0.001**	**0.004**	2.217(1.291–3.805)
Educational background: University or higher	31.4	31.9	0.007	0.007	*	
GAF mild or fewer symptoms (61–100)	64.5	49.5	5.901	0.015	–	
Employment rate	63.9	53.7	2.748	0.097	–	
Psychiatric comorbidity	27.6	31.6	0.491	0.483		
Physical comorbidity	35.1	38.9	0.394	0.530		
Suicide ideation	15.1	28.7	7.271	0.007	**0.032**	2.018(1.062–3.8347)
Psychotic symptom	8.6	16.0	3.371	0.066	-	
Personality disorder	8.1	14.7	2.971	0.085	-	
Neurodevelopmental disorder	13.0	10.5	0.352	0.553		
Alcohol or Substance abuse	5.9	9.7	1.291	0.256		
Mean ± SD			*F*			
Age (years)	46.3 ± 13.4	49.1 ± 13.6	0.105	0.107		
Age of onset (years)	31.3 ± 13.0	30.2 ± 11.0	5.23	0.479		
Years from onset(years)	14.9 ± 11.6	18.9 ± 10.3	0.046	0.00492	**0.042**	1.026(1.001–1.052)
BMI (kg/m^2^)	23.7 ± 4.2	24.5 ± 4.1	0.323	0.167		

RC; Rapid Cycling, 1–0RC; RC at the 1st and non-RC at the 2nd survey, 1-1RC; RC at the 1st and 2nd survey, GAF; Global Assessment of Functioning, OR; Odds Ratio, CI; Compatible Interval, SD; Standard Deviation. Bold values indicate the significant differences.

*GAF was not included in the logistic regression analysis.

#### 3.1.2. Outcome of patients without RC in the first survey

Univariate analysis showed that 0–1 RC had significantly higher GAF scores (*p* = 0.002) and higher rates of psychiatric comorbidity (*p* = 0.002) than 0–0 RC. Multivariate logistic regression analysis showed that 0–1 RC presented more neurodevelopmental disorders than 0–0 RC (OR = 2.779 [1.432–5.394], *p* = 0.003; Table 2).

**Table 2:** Impact of clinical characteristics of 0RC.

Variables	0–0RC	0-1RC	Stat	*p*	*p* multivariate	OR (95% CI)
	*n* = 2,284	*n* = 84				
*n* (%)			*χ* ^2^			
Female	46.7	53.6	1.53	0.216		
Bipolar I	33.5	66.5	1.29	0.304		
Educational background: University or higher	40.0	35.4	0.70	0.405		
GAF mild or fewer symptoms (61–100)	81.6	67.9	9.934	**0.002**	*	
Employment rate	67.5	66.7	0.026	0.873		
Psychiatric comorbidity	17.4	31.0	10.041	**0.002**	**	
Physical comorbidity	30.1	29.8	0.004	0.952		
Suicide ideation	9.5	14.3	2.178	0.140		
Psychotic symptom	6.8	8.3	0.313	0.576		
Personality disorder	5.3	9.5	2.893	0.089	–	
Neurodevelopmental disorder	5.6	15.5	14.113	**0.00017**	**0.003**	2.779(1.432–5.394)
Alcohol or Substance abuse	5.1	7.1	0.726	0.394		
Mean ± SD			*F*			
Age (years)	51.0 ± 13.7	48.7 ± 13.4	0.094	0.142		
Age of onset (years)	35.5 ± 12.3	32.1 ± 12.2	0.008	**0.014**	–	
Years from onset(years)	15.4 ± 10.5	16.6 ± 10.6	0.008	0.282		
BMI (kg/m^2^)	23.3 ± 4.0	24.2 ± 3.8	0.464	0.060	–	

RC; Rapid Cycling, 0–0RC; non-RC at the 1st and 2nd survey, 0-1RC; non-RC at the 1st and 2nd survey, GAF; Global Assessment of Functioning, OR; Odds Ratio, CI; Compatible Interval, SD; Standard Deviation. Bold values indicate the significant differences.

* GAF was not included in the logistic regression analysis.

** Psychiatric comorbidity was not included in Logistic regression analysis.

### 3.2. Outcome of patients with EUT

EUT was observed in 16.4% (416/2,539) of patients in the first survey, and 11.0% (279/2,539) presented EUT for two consecutive years. Of those with EUT in the first survey, 67.1% (279/416) continued presenting in the following year. Of the patients with EUT in the second survey, 50.5% (279/552) also presented in the previous year. A total of 12.9% (273/2,123) of the participants who did not present EUT in the first survey had EUT in the following year, and 72.9% (1,850/2,539) had never experienced EUT in the 2 years. Patients who had EUT at the first survey were significantly more likely to have EUT 1 year later (OR = 13.8 [10.8–17.6], *p* < 0.0001).

#### 3.2.1. Outcome of patients with EUT in the first survey

No significant differences were found between 1 and 0 EUT and 1–1 EUT in either the univariate or multivariate analyses (Table 3).

**Table 3:** Impact of clinical characteristics of 1EUT.

Variables	1–0 EUT	1-1EUT	Stat	*p*	*p* multivariate	OR (95% CI)
	*n* = 137	*n* = 279				
*n* (%)			*χ* ^2^			
Female	48.9	54.5	1.146	0.284		
Bipolar I	35.9	43.7	2.131	0.157		
Educational background: University or higher	43.9	43.3	0.015	0.903		
GAF mild or fewer symptoms (61–100)	97.1	97.8	0.221	0.638		
Employment rate	71.9	77.1	1.33	0.249		
Psychiatric comorbidity	13.9	6.5	6.238	**0.013**	*	
Physical comorbidity	32.8	33.0	0.001	0.979		
Suicide ideation	-	-	-	-	**	
Psychotic symptom	1.5	0.4	1.5557	0.212		
Personality disorder	2.2	0.4	3.236	0.072	0.106	6.508 (0.670–63.193)
Neurodevelopmental disorder	5.1	2.9	1.329	0.249		
Alcohol or Substance abuse	2.2	1.8	0.083	0.774		
Mean ± SD			*F*			
Age (years)	53.7 ± 14.1	54.9 ± 13.0	0.653	0.416		
Age of onset (years)	37.1 ± 12.6	36.4 ± 12.2	0.00002	0.627		
Years from onset (years)	16.6 ± 10.6	18.2 ± 11.7	2.716	0.18		
BMI (kg/m2)	23.3 ± 4.3	23.3 ± 3.9	0.655	0.903		

EUT; one year Euthymic, 1–0EUT; EUT at the 1st and non-EUT at the 2nd survey, 1-1EUT; EUT at the 1st and 2nd survey, GAF; Global Assessment of Functioning, OR; Odds Ratio, CI; Compatible Interval, SD; Standard Deviation. Bold values indicate the significant differences.

* Psychiatric comorbidity was not included in Logistic regression analysis.

** Logistic regression analysis could not be performed because the rate was 0% in 1EUT.

#### 3.2.2. Outcome of patients without EUT in the first survey

Univariate analysis showed that 0–0 EUT had significantly higher GAF scores (*p* < 0.0001); higher rates of psychiatric comorbidity (*p* < 0.0001), suicidal ideation (*p* < 0.0001), psychotic symptoms (*p* < 0.0001); higher rates of a coexisting personality disorder (*p* < 0.0001); younger age (*p* < 0.0001); lower age at onset (*p* < 0.0001) than 0–1 EUT. Multivariate logistic regression analysis showed that 0–0 EUT was younger (OR = 1.027 [1.017–1.037], *p* < 0.001) and had lower employment rates (OR = 1.723 [1.2071–2.337], *p* < 0.001), more psychotic symptoms (OR = 0.364 [0.175–0.754], *p* = 0.007), and more personality disorders (OR = 0.136 [0.033–0.557], *p* = 0.006; Table 4).

**Table 4:** Impact of clinical characteristics on 0EUT.

Variables	0–0 EUT	0–1 EUT	Stat	*p*	*p* multivariate	OR (95% CI)
	*n* = 1,894	*n* = 273				
*n* (%)			*χ* ^2^			
Female	43.6	48.7	2.481	0.115		
Bipolar I	34.3	34.6	0.01	0.92		
Educational background: University or higher	38.2	36	0.447	0.504		
GAF mild or fewer symptoms (61–100)	71	94.9	70.117	**<0.0001**	*	
Employment rate	63.6	72	7.176	0.007	**<0.0001**	1.723 (1.271–2.337)
Psychiatric comorbidity	23.2	10.6	22.154	**<0.0001**	**	
Physical comorbidity	31.5	28.6	0.973	0.333		
Suicide ideation	15.4	1.5	38.75	**<0.0001**	**<0.0001**	
Psychotic symptom	10.2	3	14.455	**<0.0001**	**0.007**	0.364 (0.175–0.754)
Personality disorder	8	1.1	17	**<0.0001**	**0.006**	0.136 (0.033–0.557)
Neurodevelopmental disorder	7.7	5.1	2.357	0.125		
Alcohol or Substance abuse	6.6	5.6	0.412	0.512		
Mean ± SD			*F*			
Age (years)	49.1 ± 13.3	53.5 ± 14.2	2.661	**<0.0001**	**<0.001**	1.027 (1.017–1.037)
Age of onset (years)	34.0 ± 12.2	37.9 ± 13.3	5.394	**<0.0001**	–	–
Years from onset(years)	15.1 ± 10.4	15.7 ± 11	3.161	0.402		
BMI (kg/m2)	23.4 ± 4.0	23.5 ± 4.2	0.056	0.722		

EUT; one year Euthymic, 0–0EUT; non-EUT at the 1st and 2nd survey, 0-1EUT; non-EUT at the 1st and EUT at the 2nd survey, GAF; Global Assessment of Functioning, OR; Odds Ratio, CI; Compatible Interval, SD; Standard Deviation. Bold values indicate the significant differences.

* GAF was not included in the logistic regression analysis.

** Psychiatric comorbidity was not included in Logistic regression analysis.

### 3.3. Examination of the effect of drugs on each outcome

The effect of drugs on outcomes was analyzed using propensity score matching (Table 5).

**Table 5:** The effects of each drug on each outcome were analyzed using propensity score matching.

	Li			VAP			LTG			CBZ			Antidepressant			Antipsychotic			Anxiolytic			Sleeping medicine		
	Off (%)	On (%)	*p-*value	Off	On	*p-* value	Off	On	*p-*value	Off	On	*p-*value	Off	On	*p-*value	Off	On	*p-*value	Off	On	*p-*value	Off	On	*p-*value
1-1RC	27.7	40.6	**0.024**	34.4	32.6	0.786	34	33.3	1	32	52	0.049	35.7	31.5	0.527	25.7	38.3	**0.036**	34.7	31.9	0.687	26.7	37.2	0.105
0-1RC	3.4	3.8	0.655	3.1	4.8	0.055	3.2	4.8	0.1	3.4	7.2	0.081	3.6	3.5	1	3	4.1	0.183	3	4.7	**0.035**	2.4	4.3	**0.017**
1-1EUT	62.8	70.6	0.095	68.5	62.6	0.327	67.8	63.2	0.482	66.5	78.9	0.324	68.2	64.7	0.503	69.3	63.6	0.24	66.7	68.2	0.812	69.9	64	0.211
0-1EUT	11.4	14.8	**0.022**	13.5	11.1	0.16	13.6	10.2	0.054	12.9	11.1	0.758	15.1	9.9	**0.001**	16.8	9.7	**0.001**	14.7	9.6	**0.001**	17.4	10.3	**0.001**

RC; Rapid Cycling, EUT; 1 year Euthymic, 1-1RC; RC at the 1st and 2nd survey, 0-1RC; non-RC at the first survey and RC at the 2nd survey, 1-1EUT; EUT at the 1st and 2nd survey, 0-1EUT; non-EUT at the 1st survey and EUT at the 2nd survey, Only the outcome of 1 is described, and the outcome of 0 is omitted. “On” indicates the proportion of people who were administered each drug, and “off” indicates the proportion of people who were not administered the drug. Bold values indicate the significant differences.

#### 3.3.1. Outcomes by prescription for patients with RC in the first survey

In the relationship between medications and the 1-year outcome for RC at the time of the first survey (*n* = 281), 1–1 RC received more lithium (*p* = 0.024) and antipsychotics (*p* = 0.036) than 1–0 RC. No predominant correlation was found with other medications, including antidepressants.

#### 3.3.2. Outcomes by prescription for patients without RC in the first survey

Regarding the relationship between medications and the 1-year outcome for non-RC at the time of the first survey (*n* = 2,369), 0–1 RC received more anxiolytics (*p* = 0.035) and sleeping medications (*p* = 0.017) than 0–0 RC. In the current study, antidepressants did not affect RC outcomes.

#### 3.3.3. Outcomes by prescription for patients with EUT in the first survey

There was no significant correlation between any of the drugs and the 1-year outcome for the patients with EUT at the time of the first survey (*n* = 416).

#### 3.3.4. Outcomes by prescription for patients without EUT in the first survey

In the relationship between medications and the 1-year outcome for non-EUT at the time of the first survey (*n* = 2,123), significantly more lithium was administered to 0–1 EUT than to 0–0 EUT (*p* = 0.022), whereas antidepressants (*p* = 0.001), antipsychotics (*p* = 0.001), anxiolytics (*p* = 0.001), and sleep medications (*p* = 0.001) were administered less frequently. No significant differences were observed between valproic acid, lamotrigine, and carbamazepine administrations.

## 4. Discussion

In this study, we analyzed a 2-year dataset from a large sample of patients with BD (n = 3,084) in an outpatient psychiatric clinic to examine the outcomes of RC with frequent mood episodes and EUT without mood episodes in relation to demographics, clinical characteristics, and prescribed medications. Our findings indicated that 9.7% of all patients had RC in the first year, and 4% experienced RC in both the first and second years. In the subgroup that had RC in the first year, 33.8% continued to experience RC in the second year, a rate significantly higher than the 3.5% observed in the group that did not experience RC in the first year. However, more than 60% of patients who experienced RC in the first year did not experience RC in the following year. In the Systematic Treatment Enhancement Program for Bipolar Disorder (STEP-BD), 32% of the 1,742 participants enrolled in the study experienced RC at the outset. However, a post-study survey conducted on 68% of the participants who were able to continue the study showed a significant reduction in RC incidence by 5% (23). It must be recognized that those experiencing RC might have quit the study. In our study, 2,650 (86%) of the 3,069 participants analyzed in the first survey were followed up with adequate data in the second survey. Of these, our study was able to follow-up with 80% (*n* = 263) of the 329 subjects who experienced RC in the first year through a second survey, potentially providing a more accurate depiction of their condition. Coryell et al. also reported that the percentage of RCs was approximately 19% in the first year, and then decreased to 5% between 2 and 5 years (12). Another study followed patients for 15 years and observed that four out of five cases of RC ended within 2 years of onset. These results support the idea that RC is a temporary state in the course of BD, and our study confirms this. Their study also noted that patients with RC were more likely to have onset before the age of 17 years and make serious suicide attempts. Our study similarly showed that RC had a lower age of onset and significantly more suicidal ideation (11). In terms of outcome, low age at onset is a factor that predisposes to 0–1 RC, and a long disease duration is a factor that correlates to a patient with RC continuing the presentation in the following year. The results support the idea of the earlier age of onset as a risk factor for RC and, at the same time, support the kindling hypothesis. This suggests that RC is a transient condition, but the risk of occurrence is based on the kindling hypothesis.

Numerous studies have investigated the potential of antidepressants to promote mood episodes ranging from depression to mania. In STEP-BD, antidepressants were associated with more frequent episodes; however, Coryell et al. reported that antidepressants, including tricyclics, did not contribute to switching (12). A systematic review of RC (40) did not provide clear evidence supporting that antidepressants promote manic episodes (41). In the current study, as in the systematic review, antidepressants were found to not contribute to RC outcomes. To date, the well-known risks associated with RC have been female sex and bipolar II disorder (23), but these predictors are of limited clinical utility because of their modest relationship with the frequency of mood episodes (9). Indeed, in the present study, there was no significant correlation with sex, and bipolar I disorder was found to be a risk factor. It is interesting to note that bipolar I disorder has not yet been reported as a risk factor for RC and maybe a unique feature of this disorder in Japan. Alternatively, only patients with confirmed BD were enrolled in the study; this might have reduced the enrollment of patients with bipolar II disorder, which is difficult to diagnose (14, 42–45). The GAF score was significantly lower in the 0–1 RC group than in the 0–0 RC group, which may reflect lower cognitive function and quality of life due to RC, as well as higher levels of suicidal ideation (15, 18, 23–27, 42).

A significant positive correlation between psychiatric comorbidities, especially developmental disorder comorbidities, and RC was found in the present study. Developmental disorders comprise a unique group of neurodevelopmental disorders that may affect up to 2.6% of the general population. Most patients with developmental disorders have at least one comorbid psychiatric disorder. Previous reports have also estimated the prevalence of BD in patients with autism spectrum disorder (ASD) to be 5–8%, and a study of the largest group yet in this context found that 9,062 of 700,000 children met the diagnostic criteria for ASD at the age of 16 years, with BD being six times more common in patients with ASD patients than in those without (46). In addition, a recent meta-analysis found that approximately one in 13 adults with ADHD was diagnosed with BD, and almost one in six adults with BD had ADHD (47), indicating a high comorbidity of both disorders. They also reported that patients with developmental disorders, including ADHD, developed BD at a younger age and experienced more frequent manic episodes. This may facilitate kindling and lead to more acute cycling. Although no correlation between ADHD and RC has been reported, it may be necessary to be aware of the risk of developing RC in patients with developmental disorders, especially BD with concomitant ADHD. In addition, the possibility that the diagnoses of developmental disorders and BD overlap and that the diagnoses may be artificially duplicated was investigated and ruled out (48).

In our study, RC occurrence in the previous year was a strong risk factor for RC in the following year. This has been reported in previous studies as well, with Schneck (23) reporting a history of RC as the strongest predictor of RC, and Bauer (49) reporting that patients with RC had significantly more episodes than those without in a prospective follow-up study over 12 months. These data suggest that patients who recently experienced RC are more likely to experience similar cycling in the future and require careful observation.

Significant correlates of outcomes from 0 EUT to 1 EUT were higher GAF, higher employment rate, older age, fewer psychotic symptoms, and fewer comorbid personality disorders. In the STEP-BD group, no correlations were found for age, age at onset, duration of illness, education, marital status, income, or substance use disorder. Among the factors found in this study, it was difficult to determine a causal relationship between employment rates, psychotic symptoms, and relapse rates. Meanwhile, older age and fewer comorbid personality disorders could be predictors of relapse, as these are inherent patient characteristics. Analysis of medications suggested that for patients with RC in the first-year, lithium carbonate, and antipsychotics may be risk factors for the continued presentation of RC in the following year. For subjects without RC in the first year, the prescription of anxiolytics and hypnotics may be a risk factor for RC in the following year. However, there was no correlation with antidepressants.

The Canadian Network for Mood and Anxiety Treatments (CANMAT) and International Society for Bipolar Disorders (ISBD) 2018 guidelines for the management of patients with BD state that antidepressants are not recommended for patients with RC because they have been shown to destabilize patients even when mood stabilizers are administered (50). Previous reports on antidepressant use have shown an association between RC and antidepressants in short-term retrospective studies (51–53), but long-term studies have been inconclusive (54–57). It is also noted that lithium carbonate, divalproex, and antipsychotics (olanzapine and quetiapine) are equally effective in helping patients with RC (58), while lamotrigine was not different from placebo in the maintenance treatment of patients with BD type I (59). Major studies and guidelines do not mention the effects of anxiolytics and sleeping pills in patients with BD. In this study, anxiolytics and hypnotics were negatively correlated with RC, raising the possibility that they may potentially worsen the symptoms of BD, although it is difficult to establish causality. RC has been proposed as a probable result of the ineffectiveness of lithium carbonate, and this study shows that the usage of lithium carbonate may be a risk factor for RC in the following year in patients who presently have RC. Although this is a cross-sectional observational study that cannot prove causality, this finding may be interpreted as a result of lithium carbonate and antipsychotic administration to difficult-to-treat patients who presented with RC.

In terms of EUT outcomes, lithium was associated with the following year’s EUT for patients without EUT who had some symptoms in the first year, suggesting that lithium administration may lead to symptom stabilization. In contrast, antidepressants, antipsychotics, anxiolytics, and sleeping pills were negatively correlated, indicating that the usage of these drugs may be a risk factor for relapse in the following year. Interestingly, the use of antidepressants, which have been discussed as a risk factor for relapse, showed no correlation with the risk of relapse. According to CANMAT guidelines, lithium carbonate is the first-line drug for all conditions of bipolar I disorder and maintenance therapy for bipolar II disorder, which is consistent with the present results. Several second-generation antipsychotics are first-line agents for maintenance therapy. This study suggests that antipsychotics used with the aim of euthymia may have been ineffective. As for antidepressants, the results were partially consistent with the STEP-BD results, showing that antidepressants were correlated with the number of episodes. Antidepressant treatment correlated with the outcome of relapse but not with the outcome of RC. This suggests that as the sample size of patients with RC increases, the association between antidepressant medications and the risk of RC may become more evident.

The strengths of this study include its use of data from a large sample of patients with BD over 2 years, diagnoses made by psychiatrists practicing in clinics using appropriate ICD-10 criteria, evaluation of EUT without a 1-year mood episode, which has rarely been reported as a good prognostic indicator, and use of propensity scores to minimize bias and evaluate the effect of medications on RC and EUT outcomes. It is important to mention that this was a cross-sectional study based on a questionnaire and had several limitations. First, it is difficult to identify causal relationships between factors and their respective consequences. However, certain factors present before the onset of BD, such as sex, age at onset, developmental disability, and personality disorder, may be predictive of illness trajectories. Second, it is also possible that the missing information on clinical variables influenced the results, although we tried to identify factors that were thoroughly discussed in our study group and considered clinically important and to include them in the questionnaire as much as possible. Third, this study was based on chart review rather than the use of a mood rating scale, which might have potentially affected the accuracy of symptom severity assessment. Nevertheless, attending physicians carefully reviewed the medical records including GAF scores to mitigate this possibility. Fourth, many participants received psychosocial interventions. While there are reports that the effectiveness of psychosocial therapy for patients with BD has not been established (60, 61), it has been shown to speed recovery from episodes, delay relapse, reduce residual mood symptoms, and improve psychosocial functioning when used in conjunction with pharmacotherapy (62). Information on these interventions is missing from the current study, and there may be a therapeutic bias for each patient. Fifth, in propensity score matching, SMD < 0.25 was used for balance adjustment, but some items did not meet this requirement. It is possible that the number of patients in this study was insufficient for subgroups to analyze the effect of each drug on each outcome. Future studies with larger numbers of patients are needed to confirm this finding. Finally, this study was evaluated by physicians working in 176 clinics, and an inter-rater bias might have been present. However, the majority of participating psychiatrists were certified by the Japanese Society of Neuropsychiatry and by the Ministry of Health, Labour, and Welfare as designated psychiatrists, indicating a level of expertise in psychiatric care. Therefore, it is expected that the bias in ratings between the attending physicians was relatively small.

In summary, 10.6% of patients with BDs were found to have RC in the first survey, with 3.6% experiencing RC for two consecutive years, which correlated with BP I, suicidal ideation, prolonged disease duration, increased use of lithium carbonate, and antipsychotics compared to those who experienced RC for the first year but not the second. Possible risk factors for switching to RC include comorbid developmental disabilities and the prescription of anxiolytics and sleep medications. Additionally, 16.4% of patients with BD experienced EUT in the first survey, and 11.0% experienced this state for two consecutive years. Notably, no significant correlation was observed in the patient background or medication usage between those who were asymptomatic for 1 year and those who were asymptomatic for two consecutive years. However, possible factors that might have contributed to achieving EUT included older age; a higher employment rate; fewer psychotic symptoms; fewer personality disorders; increased use of lithium carbonate, and fewer antidepressants, antipsychotics, anxiolytics, and sleeping pills compared to those who were symptomatic for two consecutive years. When comparing the first survey with 1 year of data (11) to the current two-year outcome data, it is noteworthy that the former identified conflicting factors for EUT and RC including the age of onset and developmental and personality disorders, whereas the current results revealed no shared factors affecting either phenotype concerning the outcome. Therefore, further long-term prospective studies are necessary to identify risk factors and predictors of outcomes for these conflicting conditions. The MUSUBI study is ongoing and will provide further insights into the background and course of BD.

## Data availability statement

The original contributions presented in the study are included in the article/supplementary material, further inquiries can be directed to the corresponding author/s.

## Ethics statement

This study was conducted in accordance with the Declaration of Helsinki and the Japanese “Ethical Guidelines for Epidemiological Research.” Before the study began, the research protocol was reviewed and approved by the Institutional Review Boards of the Japan Neuropsychiatric Clinics Association and the Ethics Committee of Kansai Medical University. As this study was a retrospective medical record survey, relevant information was disclosed so that patients could opt out if they so wished. A study-specific subject identification number was assigned to each patient to ensure linkable anonymity in order to protect the patients’ personal information.

## Author contributions

CT: investigation, statistical analysis, writing & editing, and visualization. MK: conceptualization, methodology, investigation, statistical analysis, writing—review & editing, visualization, and supervision. NA: resources, data curation, and sample recruitment. YK: resources, data curation, and sample recruitment. TA: resources, data curation, and sample recruitment. HU: resources, data curation, and sample recruitment. KE: resources, data curation, and sample recruitment. EK: resources, data curation, and sample recruitment. EG: resources, data curation, and sample recruitment. SH: resources, data curation, and sample recruitment. TT: investigation, methodology, and writing—review & editing. NY-F: investigation, methodology, and review & editing. AN: investigation, methodology, and review & editing. TKik: investigation, methodology, and review & editing. TKin: investigation and review & editing. KM: resources, data curation, and sample recruitment. KW: investigation, methodology, review & editing, and project administration. RY: investigation, methodology, and review & editing. All authors contributed to the article and approved the submitted version.

## Funding

This study was supported by a Ken Tanaka memorial research grant (grant numbers: 2016-2, 2017-4, and 2019-3). The funder had no role in the study design, data collection and analysis, decision to publish, or preparation of the manuscript.

## Conflict of interest

MK has received grant funding from the Japanese Ministry of Health, Labor and Welfare, the Japan Society for the Promotion of Science, SENSHIN Medical Research Foundation, the Japan Research Foundation for Clinical Pharmacology, and the Japanese Society of Clinical Neuropsychopharmacology and speaker’s honoraria from Sumitomo Dainippon Pharma, Otsuka, Meiji-Seika Pharma, Eli Lilly, MSD K.K., Pfizer, Janssen Pharmaceutical, Shionogi, Mitsubishi Tanabe Pharma, Takeda Pharmaceutical, Lundbeck, and Ono Pharmaceutical, and participated in an advisory/review board for Otsuka, Sumitomo Dainippon Pharma, Shionogi, and Boehringer Ingelheim. YK has received consultant fees from Pfizer and Meiji-Seika Pharma and speaker’s honoraria from Meiji-Seika Pharma, MSD, Eli Lilly, Janssen Pharmaceutical, Dainippon Sumitomo Pharma, Mitsubishi Tanabe Pharma, Yoshitomi Yakuhin, Otsuka Pharmaceutical, Takeda Pharmaceutical, Lundbeck Japan, and Eisai. TA has received speaker’s honoraria from Eli Lilly, Otsuka Pharmaceutical, Sumitomo Dainippon Pharma and Eisai. HU has received manuscript fees or speaker’s honoraria from Eisai Co., Ltd., Janssen Pharmaceutical, Kyowa Pharmaceutical, Meiji Seika Pharma, Otsuka Pharmaceutical, Pfizer, Shionogi, Dainippon Sumitomo Pharma, Takeda Pharmaceutical, Lundbeck Japan, and Yoshitomi Yakuhin. KE has received speaker’s honoraria from Eli Lilly, Meiji Seika Pharma, Mitsubishi Tanabe Pharma, MSD, Otsuka Pharmaceutical, Pfizer, Sumitomo Dainippon Pharma, Kyowa, Yoshitomi Yakuhin, and Takeda Pharmaceutical. EK has received speaker’s honoraria from Daiichi Sankyo, Eisai, Eli Lilly, Janssen Pharmaceutical, Kyowa Pharmaceutical, Meiji Seika Pharma, Mitsubishi Tanabe Pharma, MSD, Otsuka Pharmaceutical, Pfizer, Sumitomo Dainippon Pharma, UCB, and Viatris. SH has received manuscript fees or speaker’s honoraria from Eli Lilly, Janssen Pharmaceutical, Kyowa Pharmaceutical, Meiji Seika Pharma, Mitsubishi Tanabe Pharma, Mochida Pharmaceutical, Ono Pharmaceutical, Otsuka Pharmaceutical, Pfizer, Shionogi, Sumitomo Dainippon Pharma, Takeda Pharmaceutical, and Yoshitomi Yakuhin. EG has received manuscript fees or speaker’s honoraria from Eli Lilly, Janssen Pharmaceutical, Meiji Seika Pharma, Mitsubishi Tanabe Pharma, MSD, Otsuka Pharmaceutical, Takeda Pharmaceutical, Eisai, Ono Pharmaceutical, Kyowa Pharmaceutical Industry, and Sumitomo Dainippon Pharma. RY has received speaker’s honoraria from Eli Lilly, Dainippon Sumitomo, Otsuka, and Esai. AN has received speaker’s honoraria from Pfizer, Eli Lilly, Otsuka, Janssen Pharmaceutical, Mitsubishi Tanabe Pharma, Mochida, Dainippon Sumitomo, and NTT Docomo, and participated in an advisory board for Takeda, Meiji Seika, Tsumura, and Yoshitomi Yakuhin. TKik has received consultant fees from Takeda Pharmaceutical and the Center for Cognitive Behavioral Therapy and Training. TT has received consultant fees from Pfizer and speaker’s honoraria from Eli Lilly, Meiji-Seika Pharma, MSD, Janssen Pharmaceutical, Dainippon Sumitomo Pharma, Mitsubishi Tanabe Pharma, Yoshitomi Yakuhin, Mochida Pharmaceutical, Otsuka Pharmaceutical, Kyowa Pharmaceutical, and Takeda Pharmaceutical. KW has received manuscript fees or speaker’s honoraria from Daiichi Sankyo, Eisai, Eli Lilly, GlaxoSmithKline, Janssen Pharmaceutical, Kyowa Pharmaceutical, Meiji Seika Pharma, Mitsubishi Tanabe Pharma, MSD, Otsuka Pharmaceutical, Pfizer, Shionogi, Sumitomo Dainippon Pharma, Takeda Pharmaceutical, and Yoshitomi Yakuhin, has received research/grant support from Daiichi Sankyo, Eisai, MSD, Mitsubishi Tanabe Pharma, Meiji Seika Pharma, Otsuka Pharmaceutical, Pfizer, Shionogi, Sumitomo Dainippon Pharma, Takeda Pharmaceutical, and is a consultant for Boehringer Ingelheim, Daiichi Sankyo, Eisai, Eli Lilly, Kyowa Pharmaceutical, Lundbeck Japan, Otsuka Pharmaceutical, Sumitomo Dainippon Pharma, Taisho Toyama Pharmaceutical, Takeda Pharmaceutical, and Viatris. NY-F has received grant/research support or honoraria from, and received speaker’s honoraria of Dainippon-Sumitomo Pharma, Mochida Pharmaceutical, MSD, and Otsuka Pharmaceutical.

The remaining authors declare that the research was conducted in the absence of any commercial or financial relationships that could be construed as a potential conflict of interest.

## Publisher’s note

All claims expressed in this article are solely those of the authors and do not necessarily represent those of their affiliated organizations, or those of the publisher, the editors and the reviewers. Any product that may be evaluated in this article, or claim that may be made by its manufacturer, is not guaranteed or endorsed by the publisher.
